# Functional implications of respiratory syncytial virus F sequence variability: a comparative analysis using contemporary RSV isolates

**DOI:** 10.1128/msphere.00860-24

**Published:** 2025-04-14

**Authors:** Kim Stobbelaar, Lotte Jacobs, Francisco I. Serrano-Cano, Axelle Fransen, Winke Van der Gucht, Annemieke Smet, Benedicte Y. De Winter, Paul Cos, Winnok de Vos, Kim Van Hoorenbeeck, Stijn Verhulst, Peter L. Delputte

**Affiliations:** 1 Laboratory of Microbiology, Parasitology and Hygiene, Faculty of Biomedical Sciences, University of Antwerp 192197, Antwerp, Belgium; 2 Laboratory of Experimental Medicine and Pediatrics, Faculty of Medicine and Health Sciences, University of Antwerp 26660, Antwerp, Belgium; 3 Department of Pediatrics, Antwerp University Hospital, Edegem, Belgium; 4 Infla-Med Center of Excellence, University of Antwerp 26660, Antwerp, Belgium; 5 Department of Gastroenterology and Hepatology, Antwerp University Hospital, Edegem, Belgium; 6 Laboratory of Cell Biology and Histology, Department of Veterinary Sciences, University of Antwerp 703779, Antwerp, Belgium; 7 Antwerp Center for Advanced Microscopy, University of Antwerp 26660, Antwerp, Belgium; 8 µNeuro Research Center of Excellence, University of Antwerp 26660, Antwerp, Belgium; Icahn School of Medicine at Mount Sinai, New York, New York, USA

**Keywords:** respiratory syncytial virus, fusion protein, clinical isolates

## Abstract

Respiratory syncytial virus (RSV) remains a major global health issue. Therapeutic options are limited, but new prophylactics, all targeting the fusion (F) glycoprotein, were recently licensed. Although F sequence variation is limited, it is unclear if and how this variability translates to phenotypical differences. We analyzed full F sequences of 105 contemporary RSV isolates, gathered from children presenting with acute lower respiratory tract infections. Based on F antigenic site variation, 20 isolates were selected for functional characterization. Although RSV F diversity is generally low, the overall mean pairwise distance between RSV-A strains is higher than RSV-B (0.014 vs 0.008, *P* < 0.001). Sequence variability in the known monoclonal antibody (mAb)-binding sites seems insufficient to explain the divergent and often subgroup-dependent sensitivity to mAb neutralization. Despite comparable growth rates, mean syncytium size was higher (mean 61.68 [SD 24.51] vs 31.72 [SD 11.64], *P* < 0.001), and mean syncytium frequency was lower (mean 25.37 [SD 9.20] vs 47.85 [11.52], *P* < 0.001) in RSV-A strains. For all isolates, higher temperatures were associated with increased inactivation, although some RSV-A isolates displayed manifestly higher stability. Genomic and phenotypic variability among our contemporary RSV isolates was limited, with noticeable exceptions. The availability of new prophylactics targeting RSV F corroborates the importance of continued RSV surveillance to identify changes in the F sequence, including mutations that reduce mAb efficacy or lead to escape mutants. Surveillance should include both sequencing data and an evaluation of sensitivity to prophylactic antibodies, using contemporary clinical isolates.

**IMPORTANCE:**

Respiratory syncytial virus (RSV) is a major cause of respiratory infections in young children worldwide. Recent progress has led to new ways to prevent serious RSV-associated disease. The virus’s fusion (F) protein is a key focus for vaccine development because it helps the virus enter host cells and is well conserved across different virus strains. However, it is unclear if small differences in the F protein sequence could affect how the virus behaves *in vitro*. In this study, we, therefore, analyzed 105 RSV samples from children under two who presented with respiratory infections. We selected 20 samples (12 RSV-A and 8 RSV-B) for functional testing, based on their F protein sequences. Phenotypic differences between clinical isolates and reference strains, such as virus stability at 4°C and susceptibility to monoclonal antibody neutralization, highlight the importance of using viruses isolated from recent clinical samples. Although significant functional differences were observed in traits related to the F protein, both between the RSV subgroups and within, the underlying molecular mechanisms remain unclear. Ongoing monitoring of RSV is critical to ensure current and future vaccines remain effective.

## INTRODUCTION

Respiratory syncytial virus (RSV) represents the primary cause of acute lower respiratory tract infections (ALRTI) in children under the age of five. In 2019, an estimated 33.0 million episodes of RSV-associated ALRTI occurred globally (ranging from 25.4 to 44.6 million), leading to 3.6 million hospital admissions (2.9–4.6 million) and 101,400 RSV-attributable deaths (84,500–125,200) in this age group alone (1). The clinical presentation of RSV in children varies widely, ranging from mild symptoms, mimicking a common cold, to severe ALRTI necessitating invasive ventilation. Although young age (<6 months), prematurity, and the presence of underlying comorbidities or immunodeficiency are independent risk factors for hospitalization, the majority of severe cases requiring hospital admission occur in otherwise healthy children (2, 3).

RSV is a single-stranded, non-segmented, negative-sense RNA virus, belonging to the *Orthopneumovirus hominis* species within the Pneumoviridae family (4). It has two antigenic subgroups, RSV-A and RSV-B, that cocirculate simultaneously each year, with alternating predominance. Below the subgroup level, 24 different lineages exist within RSV-A and 16 within RSV-B (5). The RSV genome consists of 10 genes that encode for 11 proteins. Of these, the attachment (G) and fusion (F) proteins, both located in the viral envelope, are the most important due to their essential roles in viral fusion and entry. Moreover, they are the only RSV proteins eliciting neutralizing antibodies (6). The G protein is, however, less conserved and was shown to be dispensable for viral replication, making G a less attractive target in vaccine and monoclonal antibody (mAb) research, as opposed to F (7, 8). RSV F is initially translated as an inactive F0 precursor, which undergoes dual cleavage by furin-like host proteases to achieve a fusion-competent state. Following trimerization, the mature F protein facilitates the fusion of viral and host cell membranes, transitioning from a metastable prefusion conformation to a highly stable postfusion conformation. RSV F harbors various conformation-dependent antigenic sites, of which the pre-F specific sites Ø and V are considered the most neutralizing (9).

While the virus was first identified in 1956, effective and safe antivirals are still lacking, and the treatment for RSV infections is, therefore, still primarily supportive in nature, involving the supplementation of oxygen or fluids as needed (10). Historically, the only preventive measure available was mAb palivizumab, which is reserved for high-risk children only because of its high cost and requirement for repeated intramuscular administration (11). However, in recent years, there has been significant progress in the development of RSV prophylactics. In 2023, the first-ever RSV vaccines designed to protect both elderly (Arexvy and Abrysvo) and young infants through maternal immunization (Abrysvo) were approved (12–14). This year, a third vaccine was licensed for use in older adults (mResvia) (15). Additionally, a new mAb, nirsevimab, with an extended half-life, is now available for the protection of all children during their first RSV season (16). These new and much-anticipated prophylactics are expected to significantly alter the RSV landscape.

Given these new developments, sustained surveillance of RSV epidemiology and biology is imperative. It will be critical to monitor how these new prophylactics might induce changes in the viral genome, which could translate to a phenotype that might affect mAb and vaccine effectiveness. While virus genome-based surveillance networks are already implemented, the link between the viral genome and the functional phenotype remains incomplete. Furthermore, most of our knowledge about RSV stems from historical and potentially laboratory-adapted RSV strains, underscoring the urgent need to acquire and characterize recent clinical RSV isolates. Here, we present a detailed characterization, with an emphasis on the RSV F protein, of a repository of over 100 clinical RSV isolates, collected over the past 6 years in a tertiary hospital in Antwerp, Belgium.

## MATERIALS AND METHODS

### Patient inclusion, clinical sampling, and data collection

In this single-center study, which is part of the Respiratory Virus Repository Antwerp project, previously healthy children aged 28 days to 2 years with medically attended ALRTI were prospectively enrolled at Antwerp University Hospital, Edegem, Belgium, between October 2017 and April 2023. This included patients seen during an outpatient visit, as well as patients admitted to the general pediatric ward or pediatric intensive care unit (PICU). Patients with symptomatic cardiopulmonary disease or immunodeficiency were excluded from the study to ensure accurate clinical comparisons, as their inclusion could confound the results. Given that RSV is the primary etiological agent of interest in this study, only patients with an unknown or positive diagnostic PCR result for RSV were eligible for inclusion. Patients who had a prior negative PCR result for RSV were not considered for participation. Written parental informed consent was registered before inclusion.

After inclusion, nasal secretions were collected by nasopharyngeal aspiration via a standardized procedure: 2 mL of physiologic saline solution was injected into one of both external nostrils, and this solution was aspirated through a flexible rubber hose in a closed suction system. As nasopharyngeal swabs became the standard of care after 2021, nasopharyngeal sampling was performed with swabs from then onward by inserting the swab in one nostril until the nasopharynx was reached and then rotating three times. Swabs were stored in a container prefilled with 0.9% saline. Immediately after sample collection, the samples were preserved at 4°C and transferred to the lab for further processing.

Parents as well as the responsible physician were asked to complete a questionnaire on basic demographic and clinical data. These data were used to determine disease severity for each included patient, following the ReSViNET scale (17). Additionally, the length of the hospital stay and the need for supplemental oxygen administration and PICU admission were recorded as a proxy for disease severity.

### Virus isolation and propagation

Upon arrival at the lab, the nasopharyngeal sample was divided into different aliquots, each containing approximately 200 µL. All but two aliquots were immediately snap frozen after a 1:1 dilution in Hanks’ balanced salt solution containing 40% sucrose, using a mixture of dry ice and 99% ethanol, and stored at −80°C. One aliquot was retained at 4°C for RNA extraction and subsequent confirmation of RSV infection. The remaining aliquot was processed in a serial 1:4 dilution in cell culture medium and inoculated onto a 96-well plate with 80% confluent monolayers of HEp-2 cells for virus isolation. Following a 7-day incubation period at 37°C and 5% CO_2_, the process was repeated twice, each time selecting eight wells with the highest virus dilution that continued to show a distinct cytopathic effect (CPE). In the third inoculation round, viral supernatants of five CPE-positive wells (250 µL) were transferred to a pre-seeded culture flask, which was harvested by scraping after 2–5 days and subsequently centrifuged, snap frozen, and stored at −80°C. Reference viruses RSV-A2 and RSV-B1 were obtained from Biological and Emerging Infections (BEI) resources. For functional analysis, passage 3 (P3) isolates were used. Viral titers were calculated using a conventional plaque-forming unit (PFU) assay, in triplicate, as described elsewhere (18).

### Cells

All virus isolates were propagated in sub-confluent HEp-2 cells, apart from the RSV-B1 reference strain, which is routinely cultivated in Vero cells. One RSV-B clinical isolate was also grown on Vero cells, as viral titers after propagation on HEp-2 cells appeared insufficient for functional testing. HEp-2 and Vero cell lines were acquired from ATCC and cultured in Dulbecco’s modified Eagle medium (DMEM; Gibco) containing 10% inactivated fetal bovine serum (iFBS; Gibco) and 1% penicillin-streptomycin (Gibco).

### Sequencing of G and F genes

Viral RNA was extracted from 200 µL of infected cell culture supernatants using the Maxwell RSC Viral Total Nucleic Acid Purification Kit and the Maxwell CSC Instrument, according to the manufacturer’s instructions. After quality and quantity determination with Nanodrop equipment, reverse transcription and PCR amplification were performed on a thermal cycler using the one-step RT-PCR kit (Qiagen) and primers for the G and F gene, described by Tapia et al. (19). Sanger sequencing was subsequently performed by the VIB Neuromics Support Facility (University of Antwerp). SnapGene (https://www.snapgene.com/) and BioEdit v7.2.5 software (20) were ultimately used for constructing consensus sequences.

### Phylogenetic analysis and assessment of F sequence variability

For phylogenetic analysis, full sequences of both G and F were used, as described by Goya et al. and the RSV Genotyping Consensus Consortium in their recently proposed global consensus (5). Sequences were assigned to the existing lineages using the online tool NextClade (https://clades.nextstrain.org/). Once our sequences were translated *in silico* and aligned with reference sequences from Goya et al. using MAFFT (21), and maximum likelihood phylogenetic trees were inferred with IQ-TREE v2.2.0 (22) and visualized with Figtree v1.4.4 (http://tree.bio.ed.ac.uk/software/figtree/). Pairwise distances and overall mean distances were calculated using MEGA X: Molecular Evolutionary Genetics Analysis version 10 for MacOS (23).

Additionally, allele frequencies were calculated at each amino-acid residue. Cumulative minor allele frequency (MAF) at each residue was defined as the sum of the allele frequencies of all individual variants, excluding the major allele, within the clinical isolates. Sites having a cumulative MAF of ≥3% were considered polymorphic. For each sample, the mean cumulative MAF was calculated by dividing the cumulative MAF at each residue by the total amount of residues, analogous to Lin et al. (24). Of those samples with a high mean cumulative MAF (i.e., higher than the median), a selection was made for functional *in vitro* testing. Some isolates had a rather low mean cumulative MAF but contained variants in coding sequences of known antigenic sites and were therefore also included. This led to a panel of 12 clinical RSV-A isolates and 8 clinical RSV-B isolates for subsequent phenotypical analysis.

### Growth kinetics assessment

To evaluate infectious virus production at different timepoints post-infection (p.i.), 24-well plates containing 90% confluent monolayers of HEp-2 cells were infected with a multiplicity of infection (MOI) of 0.01 of both the clinical isolates and the reference strains RSV-A2 and RSV-B1. Reactions were performed in triplicate. After 2 h of incubation at 37°C and 5% CO_2_, the inoculum was removed and replaced by DMEM containing 10% FBS. At 24, 48, and 72 h p.i., cell monolayers were scraped into supernatant without lysis of cells, pelleted by centrifugation at 1,000 × *g*, and snap frozen. Supernatants of the different tested timepoints were subsequently titrated with a conventional PFU assay.

### Thermal stability assay

For each virus isolate and the reference strains, a virus suspension of 1 × 10^5^ PFU/mL or 1 × 10^4^ PFU/mL was created in DMEM without serum for RSV-A and RSV-B strains, respectively. Different starting concentrations were used because of important differences in viral titers between subgroup A and B. The virus suspensions were divided into different aliquots that were stored at 4, 32, or 37°C for 24, 48, and 72 h. Immediately after suspension and after each of the indicated timepoints, three aliquots per condition were snap frozen and stored at −80°C until titration by conventional PFU assay.

### Fusogenicity assay

The fusion capacity of the different isolates was evaluated as described in Van der Gucht et al. (18). Briefly, 24 h prior to inoculation, HEp-2 cells were seeded at a concentration of 17,500 cells/well in black CELLSTAR 96-well plates with a µclear flat bottom (Greiner Bio-One). These cells were then inoculated with both clinical RSV isolates and the RSV reference strains RSV-A2 and RSV-B1 at an MOI of 0.05, using three biological replicates. After an incubation of 2 h at 37°C and 5% CO_2_, the inoculum was removed and replaced by DMEM 10% containing 0.6% Avicel (FMC BioPolymer). After 48 h, immunofluorescent staining of infected cells was performed. To this end, cells were fixed with 4% paraformaldehyde (PF) solution (Merck KGaA), permeabilized with Triton X-100 (Perkin Elmer), and stained with palivizumab (Synagis, Astra Zeneca), followed by a goat-anti-human secondary antibody conjugated with AF488 (Invitrogen). 4',6-Diamidino-2-phenylindole (DAPI) (Sigma-Aldrich) was used to stain the nuclei (25). Images were acquired on a Nikon Ti wide-field fluorescence microscope (Nikon Instruments, Paris, France) using a 10× dry lens (numerical aperture 0.3). For each biological replicate, three well or technical replicates were analyzed. Per well, five frames were acquired in two channels (395 and 470 mm excitation). Image processing was performed in Fiji, a packaged version of ImageJ freeware (https://fiji.sc) using the CellBlocks.ijm (v.20) script (26, 27), which was extended for plaque detection and is available on Github (https://github.com/DeVosLab/CellBlocks). In brief, nuclear objects were detected using the StarDist trained classifier (probability 0.15; overlap tolerance 0.3), and only objects below 50 µm^2^ in size were excluded. This strategy ensured that both single nuclei and fused nuclei would be detected. Plaques were detected in the AF488 channel by setting a fixed threshold and retaining all objects with a size larger than a single nucleus. Subsequently, the average size of syncytia was determined as well as their frequency.

### Neutralization assay with a panel of different mAbs

A neutralization assay was performed in HEp-2 cells as described by Leemans et al. (25). Different mAbs targeting different antigenic sites of the RSV F protein were evaluated (Table 1). All mAbs were purchased from Cambridge Biologics, LLC (Brookline, MA, USA), except for palivizumab, which was acquired as leftovers from the Antwerp University Hospital. The choice was made not to include a post-F-specific site I mAb, as these are considered weakly neutralizing. For each mAb that was tested, a 1:2 dilution series was made in DMEM, starting from a concentration of 3.12 µg/mL. The lower limit of detection (LOD) was 0.05 µg/mL. All RSV isolates were diluted to a virus suspension containing 1,200 PFU/mL. This suspension was subsequently added to the mAb dilution series and allowed to interact at 37°C for 1 h. Hereafter, the mAb-virus suspension was added to a pre-seeded 96-well plate and incubated for another 2 h. After this incubation period, the inoculum was removed, and a 1:4 mixture of 2.4% Avicel in DMEM 10% was added to the infected cells. The plates were then incubated for 72 h, after which they were fixed with 4% PF, permeabilized with Triton X-100, and stained with palivizumab and a secondary goat anti-human horseradish peroxidase (HRP)-conjugated antibody (Invitrogen). Chloronaphtol (Thermo Fisher Scientific) was used to visualize the plaques. The mAb titer leading to a 50% reduction in viral multiplication, expressed as IC50, was subsequently determined by manual counting. Each analysis was performed in triplicate, with non-neutralized virus serving as a control.

**TABLE 1:** List of anti-RSV F mAbs used in the neutralization assay, detailing their respective antigenic sites and targeted conformation of the F protein

Monoclonal antibody	Antigenic site	RSV F protein conformation
D25	Ø	Prefusion
MEDI8897 (nirsevimab)		
AM22		
5C4		
MEDI493 (palivizumab)	II	Prefusion and postfusion
MPE8	III	Prefusion
101F	IV	Prefusion and postfusion
AM14		
CR9501	V	Prefusion
hRSV90 (suptavumab)		

### Association of disease severity and other viral traits with amino acid usage at each RSV F residue

The meta-CATS algorithm (28) was used to identify amino acid (AA) residues within the RSV G and F protein that were statistically associated with disease severity, as expressed by the ReSViNET grade (17) and the need for supplementary O_2_ or PICU admission. Protein sequences were aligned using MUSCLE. Analysis was performed separately for each subgroup.

### Statistical analysis

Demographic data are expressed as either frequencies, for dichotomous categorical variables, or medians with interquartile range (IQR). Normality testing of continuous variables was performed using the Shapiro-Wilk test. Data from MAF analysis, fusogenicity assay, and neutralization assays were analyzed with the Kruskal-Wallis rank sum test and Wilcoxon rank test for post-hoc pairwise comparison when comparing individual samples, and the Wilcoxon test for comparison between the two subgroups. To test for differences in growth kinetics between individual samples and between subtypes, a linear mixed model was fitted to log-transformed data. The dependence between observations from the same individual sample and same biological replicate (nested within the sample) was accounted for. The fixed effects included timepoint, subgroup, and the interaction between them. For the thermal stability assessment, just like for the growth kinetics data, linear mixed models were fitted on log-transformed data to model the evolution of PFU over time, accounting for the dependence between observations for the same individual sample. However, as technical replicates were analyzed here, no batch effects were accounted for. The model included a random slope and a random intercept. The level of significance was set to *P*-values below 0.05. Statistical analyses were conducted in R version 4.1.1 (2021-08-10) (29), and figures were produced using the package ggplot2 (30).

## RESULTS

### Study population

Over the course of five RSV seasons, a total of 174 infants and young children suffering from a medically attended viral ALRTI were enrolled in this study. After nasopharyngeal sampling, 155 (89%) of these patients were determined to be positive for RSV by qPCR. Virus isolation was subsequently attempted, leading to 105 (68%) successful RSV isolates, with 52% (55/105) of samples belonging to the RSV-A subgroup and 48% (50/105) belonging to the RSV-B subgroup (Table 2). General and clinical characteristics of patients from whom RSV was successfully isolated are detailed in Table 3. The median age of these patients was 5.59 months (IQR 1.94–11.00), and 94% of these RSV-positive patients were hospitalized, with a median hospital stay of 5.00 days (IQR 3.00–7.00).

**TABLE 2:** Summary of the number of samples that tested positive for RSV, were successfully propagated, or were selected for functional testing, categorized by the season of inclusion^*a*^

		Total	2017–2018	2018–2019	2019–2020	2021–2022	2022–2023
Number of RSV-positive samples	RSV-A	78	11	10	31	16	5
	RSV-B	82	11	38	32	0	1
Number of successfully propagated samples	RSV-A	55	8	5	24	15	3
	RSV-B	50	4	25	20	0	1
Number of samples selected for functional testing	RSV-A	12	4	0	4	2	2
	RSV-B	8	3	3	1	0	1

^*a*^ All samples collected during the 2019–2020 season were collected before the COVID-19 pandemic. No samples were collected during the 2020–2021 season.

**TABLE 3:** Basic characteristics of 105 patients with successful RSV isolates^*a*^

Demographics	All (*N* = 105)	RSV-A (*N* = 55)	RSV-B (*N* = 50)
Sex, *n* (%)			
Male	70 (67)	35 (64)	35 (70)
Female	35 (33)	20 (36)	15 (30)
Age at inclusion, months (median, IQR)	5.59 (1.94–11.00)	5.96 (1.81–10.78)	5.56 (2.46–11.29)
Gestational age, *n* (%)			
Term	93 (89)	50 (91)	43 (86)
Preterm (<37 weeks)	12 (11)	5 (9)	7 (14)
Disposition type, *n* (%)			
Outpatient	6 (6)	3 (5)	3 (6)
General pediatric ward	74 (70)	34 (62)	40 (80)
PICU	25 (24)	18 (33)	7 (14)
Disease severity indices			
ReSViNET grade, *n* (%)			
Mild (0–7)	27 (26)	14 (25)	13 (26)
Moderate (8–13)	65 (62)	36 (65)	29 (58)
Severe (14–20)	13 (12)	5 (9)	8 (16)
Length of hospital admission, days (median, IQR)	5 (3–7)	5 (3–8)	4 (3–6)
Supplemental oxygen need, *n* (%)	72 (69)	37	35

^*a*^ This table summarizes the demographic and clinical data of the patient cohort, as well as various indices of disease severity.

### Phylogenetic analysis revealed greater genetic diversity within the RSV-A subgroup

RSV-B strains from our collection displayed limited variation, with an overall mean pairwise distance of 0.008 (SD 0.004). Most isolates belonged to the B.D.4.1.1 lineage, harboring the lineage-defining I206M and Q209R mutations within site Ø in the heptad repeat A region of the F protein (Fig. S1). Three RSV-B isolates were classified under the descendant lineage, B.D.E.2, two belonged to the parental lineage B.D.4.1, and two isolates seem to cluster out of any of the known clades. Our RSV-A strains had a significantly higher overall mean pairwise distance of 0.014 (SD 0.008, *P* < 0.001). The most prevalent lineage was A.D.5, with 53% of strains assigned to this or one of the nested lineages, although several other lineages, such as A.D and its descendant lineages A.D.1–4, were also represented. (Fig. S2). It should be noted that most of the included RSV-B isolates date from the pre-COVID-19 period, and only 1 B strain was isolated after the COVID-19 pandemic (Table 2), which might explain the lower mean pairwise distance observed here.

### The F-coding sequence is highly conserved in the different antigenic sites

**Fig 1:**
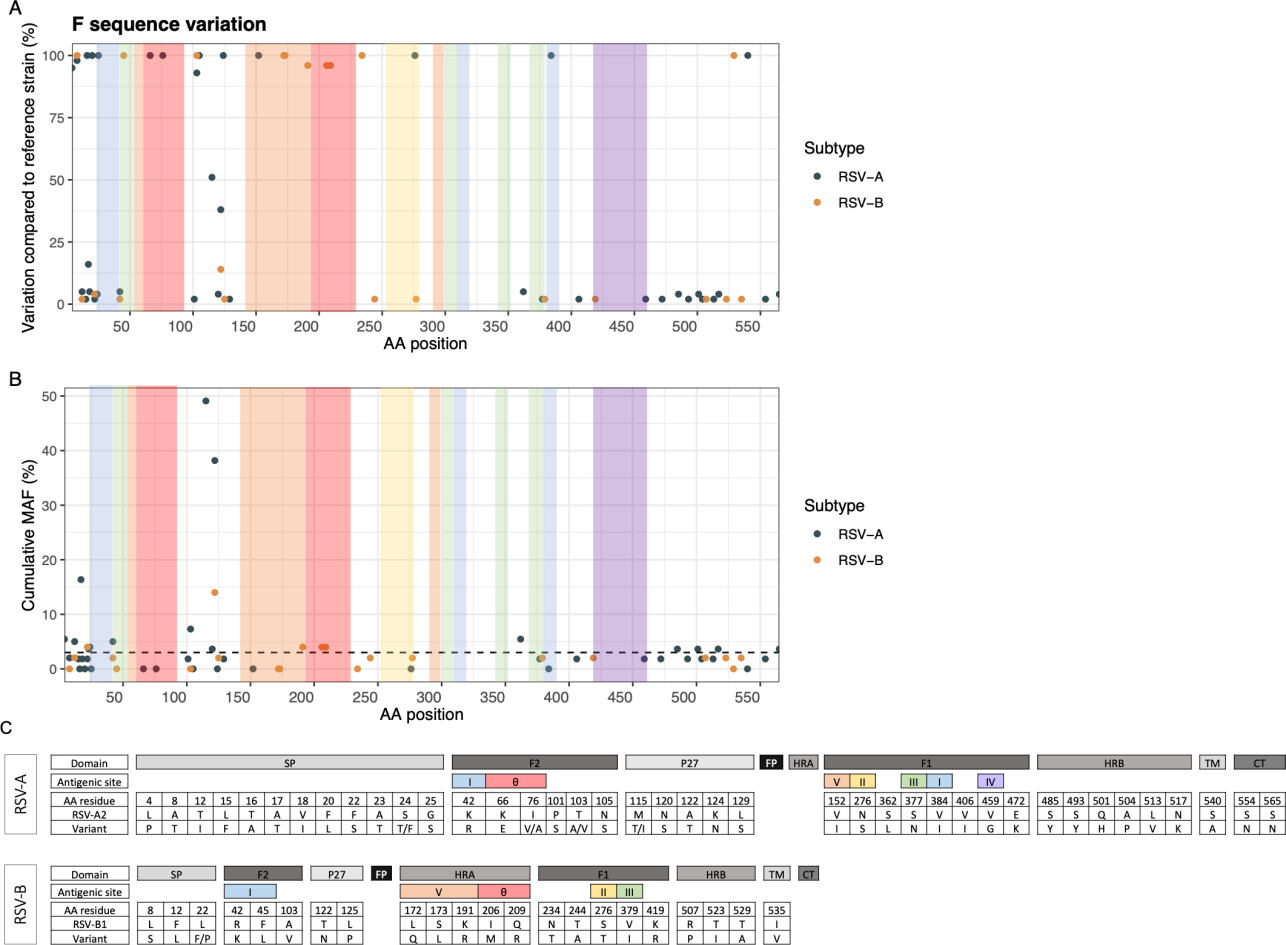
Frequency and distribution of variants in the coding regions of the F sequence. Frequencies are expressed as the percentage of samples with a variant compared to the respective reference strain (**A**) and as cumulative MAF (**B**) at each AA residue. The dashed line in panel B indicates the 3% threshold for polymorphic residues. Panel C shows the different variants at each residue, compared to the respective reference strain. Colored panels represent the different antigenic sites: Ø (red), I (blue), II (yellow), III (green), IV (purple), and V (orange).

A multiple sequence alignment of F protein AA sequences of all 55 RSV-A and 50 RSV-B isolates, together with their respective reference strains, RSV-A2 and RSV-B1, revealed higher variability in RSV-A compared to RSV-B, mainly in the signal peptide, the p27 peptide, and heptad repeat B (HRB) domains of F (Fig. 1A). Similarly, the mean cumulative MAF per sample differs significantly between both subgroups, with a mean of 0.33% (SD 0.25) for RSV-A strains vs 0.09% (SD 0.19) for RSV-B strains (*P* < 0.001). For both subgroups, cumulative MAF was highest in the p27 peptide with mutations in position 122 leading to the addition or loss of a N-glycosylation site in RSV-A or RSV-B samples, respectively (Fig. 1B).

Overall, the AA sequences of the different antigenic epitopes of the F protein were highly conserved in our study. All RSV-B samples contain the L172Q/S173L substitutions in antigenic site V, associated with the notorious suptavumab failure (31). Most RSV-B samples, except two isolates from 2017, contained two mutations in the nirsevimab-binding site in antigenic site Ø (I206M and Q209R), compared to the RSV-B1 reference strain. However, these mutations have not been associated with reduced sensitivity to nirsevimab. Additionally, a mutation in antigenic site V, K191R, seems associated with these mutations in site Ø, following the same distribution. All samples from both subgroups carry a serine at residue 276, which is adjacent to the palivizumab-binding site in antigenic site II, apart from one RSV-B isolate with an S276T mutation. The importance of this residue in resistance to palivizumab has, however, already been questioned (32–34). The specific binding sites for the other mAbs included in this study (Table 1) demonstrated 100% sequence conservation, although some antigenic sites showed AA substitutions at low frequencies, such as V76A (1/55 RSV-A) in site Ø, R42K (1/50 RSV-B) and K42R (3/55 RSV-A) in site I, S377N (1/55 RSV-A) and V379I (1/50 RSV-B) in site III, and V459G (1/55 RSV-A) in site IV.

To assess adaptation to cell culture conditions, we compared the F protein sequences between P0, the first passage of isolates on HEp-2 cells in a culture flask, and P3 isolates. For three isolates, insufficient material remained from the P0 passage to conduct this analysis. In 11 samples, the sequences were identical between both passages. In five samples, we identified a single amino acid substitution in the signal peptide (L22P), antigenic site I (R42K), p27 peptide (P101S), antigenic site IV (W439C), and HRB (T508R). One sample exhibited two mutations in HRB (T523I and E533V). Notably, only one of these residues, W439C, is located in proximity to the binding sites of the tested mAbs 101F and AM14.

### Correlation between F sequence variability and clinical disease severity

Comparative analysis using the meta-CATS service to identify amino acid positions potentially correlating with clinical severity, as quantified by the ReSViNET-scoring system, revealed significant differences between mild (grade I) and severe (grade III) patients at nucleotide position 537 in the G protein (T vs C, *P* = 0.02) and nucleotide position 1,683 in the F protein (C vs T, *P* = 0.02), within the RSV-A subgroup. However, these positions corresponded to synonymous substitutions, thus not affecting the AA sequence. No significant differences in G or F sequence were observed for RSV-B. Similarly, no correlation between sequence variability and oxygen supplementation requirements or the need for PICU admission was detected for either subgroup.

### Differences in growth kinetics and fusion capacity are larger between subgroups than within

**Fig 2:**
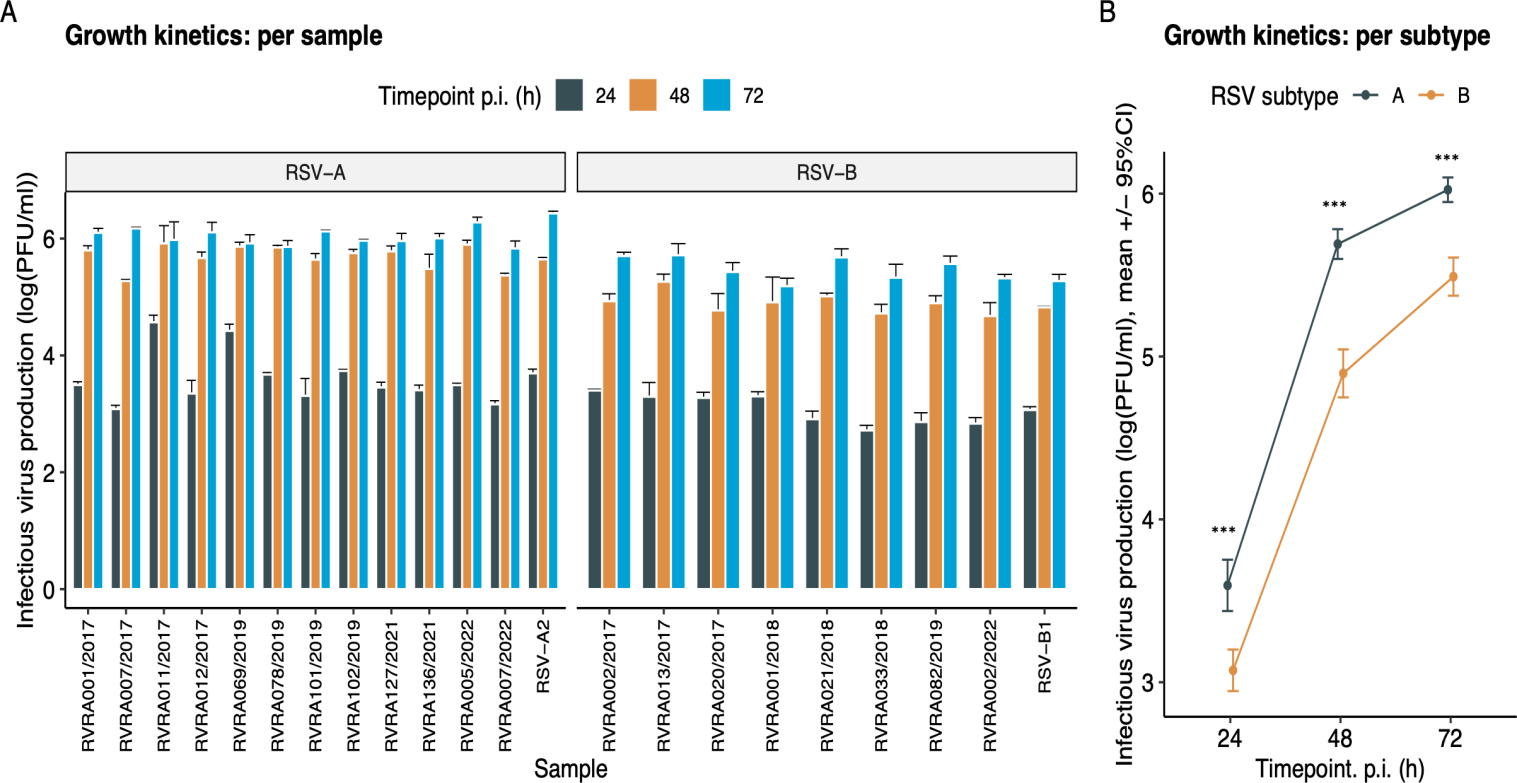
Growth kinetics of different RSV isolates, expressed as infectious virus production at each timepoint. Results are shown per sample (*n* = 22) (**A**) and per subgroup (*n* = 20) (**B**). Reference strains are not included in the subgroup analysis. Data are log transformed. A linear mixed effects regression was run to evaluate growth velocity. Differences in viral titers at a certain timepoint were assessed with the Kruskal-Wallis test for the per sample analysis and the Wilcoxon test for the per subgroup analysis. Error bars represent three biological replicates per sample. Significant differences between subgroups at each timepoint are indicated by * for *P* < 0.05, ** for *P* < 0.01, and *** for *P* < 0.001. Growth rate did not differ significantly between subgroups (*P* = 0.95).

To evaluate potential differences in the growth kinetics of RSV isolates, infectious virus production was evaluated at different timepoints post-infection. Important differences were observed between the two subgroups, with all RSV-A strains reaching titers of 10^6^ PFU/mL within 72 h, whereas none of the tested RSV-B strains reached this titer within the same time frame (Fig. 2). Although clinical RSV-A strains exhibited significantly higher quantities of infectious virus particles at each individual timepoint compared to RSV-B strains (*P* < 0.001 for each timepoint), a linear mixed effects regression was run on log-transformed data and indicated no significant difference in growth kinetics between clinical isolates of both subgroups (*P* = 0.95; Fig. 2B). Notably, isolates HRSV/A/BEL/RVRA011/2017 and HRSV/A/BEL/RVRA069/2019 demonstrated particularly high viral titers at 24 h, compared to the other RSV-A isolates and the RSV-A reference strain, but this difference was non-significant (Fig. 2A).

**Fig 3:**
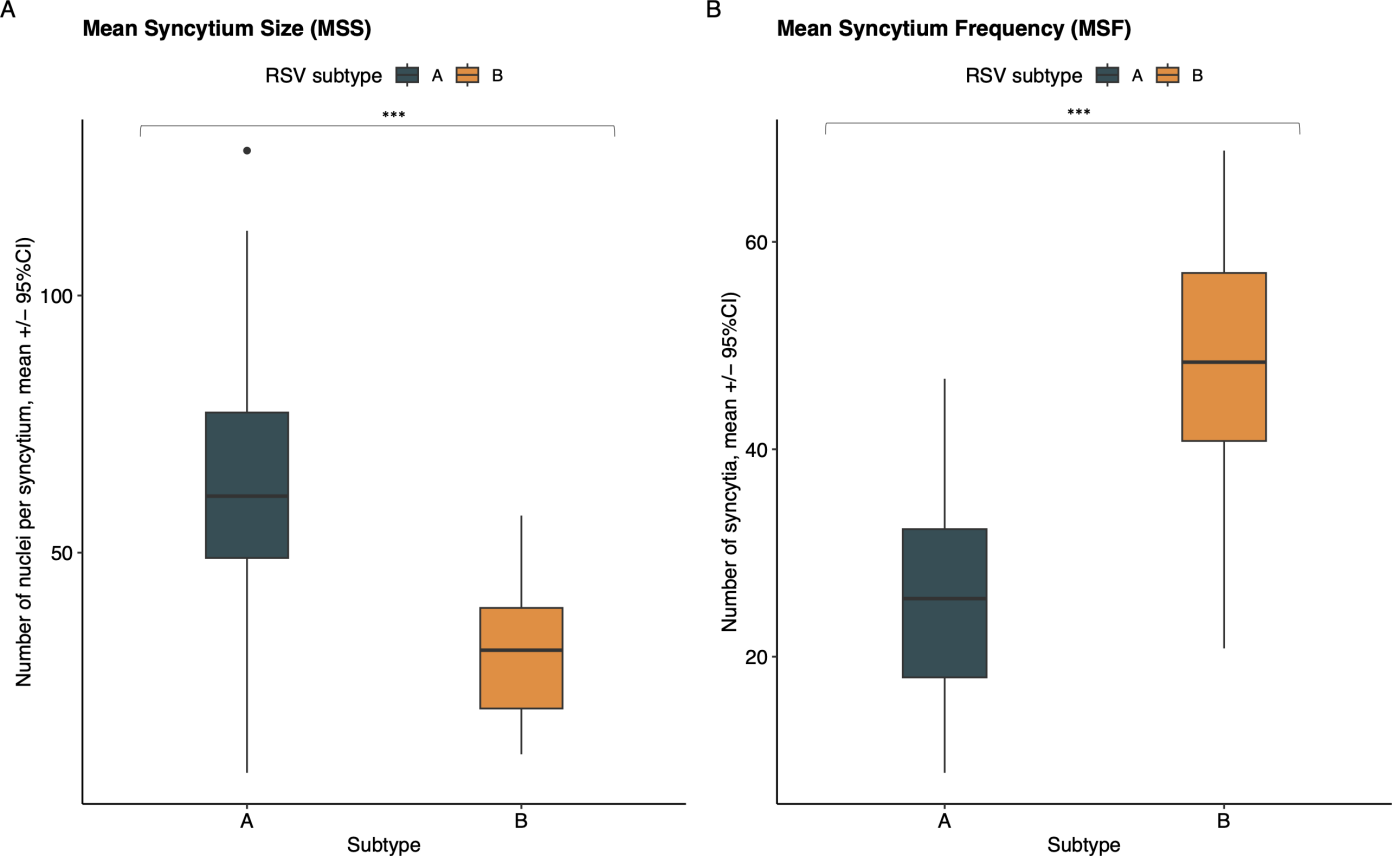
Comparison of MSS (**A**) and MSF (**B**) between the two subgroups. Data are expressed as mean ± 95% CI. The Wilcoxon test was used for subgroup comparisons. Three biological replicates were analyzed for each isolate. Significant differences are indicated by * for *P* < 0.05, ** for *P* < 0.01, and *** for *P* < 0.001.

One of the main functions of the RSV F protein is to mediate fusion during viral entry. In infected cells that express F on the cell surface, this also leads to syncytia formation. The fusion capacity of our isolates was therefore assessed by measuring mean syncytium size (MSS) and mean syncytium frequency (MSF; Fig. 3). The MSS and MSF of the individual samples can be found in the supporting information (Fig. S3). While MSS was significantly larger in clinical RSV-A isolates (mean 61.68 [SD 24.51] vs 31.72 [SD 11.64], *P* < 0.001; Fig. 3A), MSF was significantly lower (mean 25.37 [SD 9.20] vs 47.85 [11.52], *P* < 0.001; Fig. 3B), compared to RSV-B strains. Looking at each subgroup individually, two RSV-A strains, HRSV/A/BEL/RVRA069/2019 and HRSV/A/BEL/RVRA078/2019, exhibited significantly lower MSS and MSF (Fig. S3). Sequence analysis of these isolates, in comparison to the other RSV-A strains, revealed no AA differences between these two isolates and any of the others. Within the RSV-B subgroup, the HRSV/B/BEL/RVRA013/2017 and HRSV/B/BEL/RVRA020/2017 demonstrated significantly lower MSS compared to the other RSV-B strains. These isolates both lack the I206M and Q209R mutations present in the other RSV-B isolates. The RSV-B1 reference strain, however, also does not contain these two mutations and is associated with significantly larger syncytia (Fig. S3).

**Fig 4:**
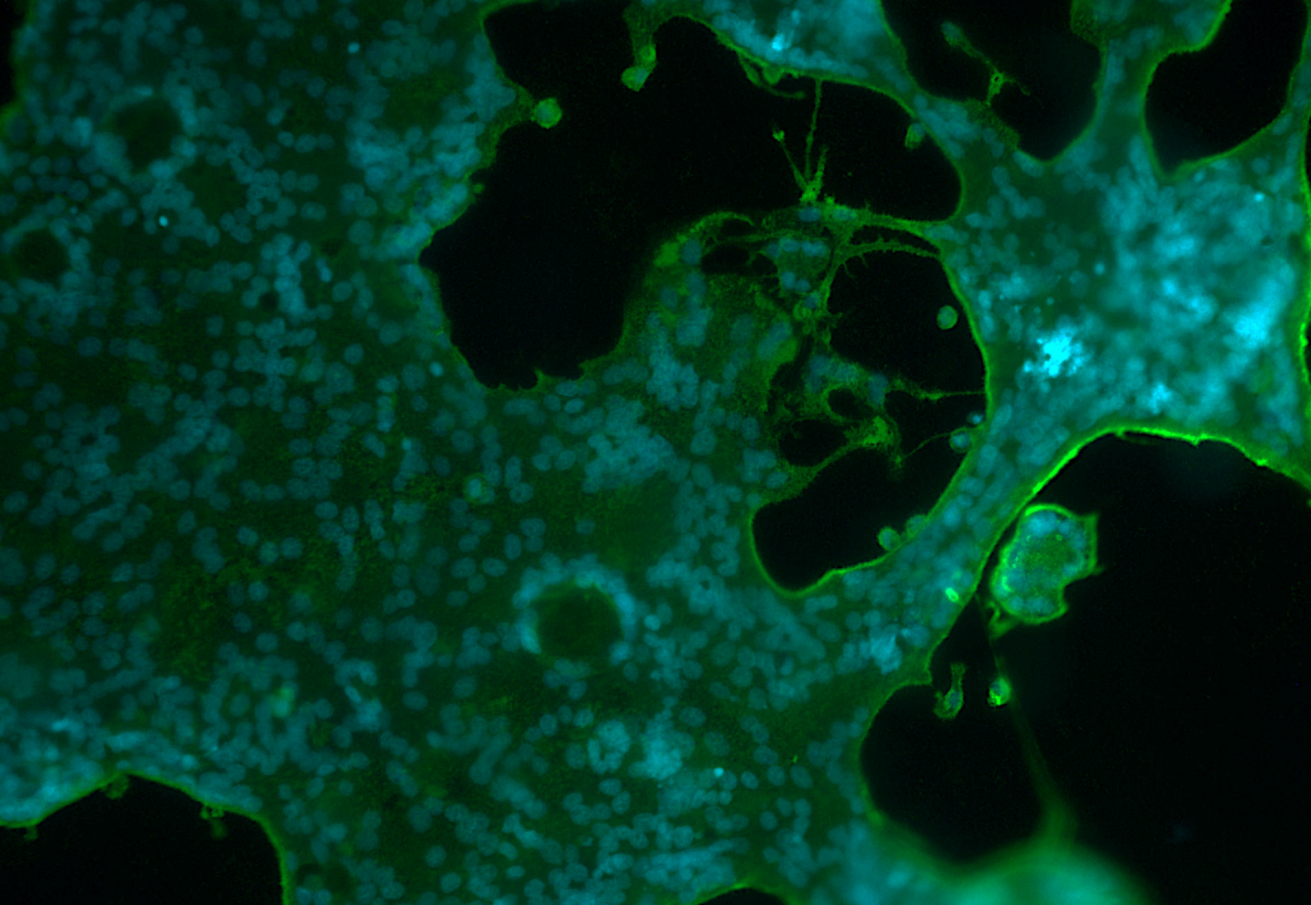
Immunofluorescent image (10× magnification) of a giant syncytium formed by the HRSV/A/BEL/RVRA011/2017 isolate. Nuclei are stained with DAPI (blue), and RSV-infected cells are stained with palivizumab, followed by a secondary goat anti-human AF488-labeled antibody (green).

One RSV-A sample, HRSV/A/BEL/RVRA011/2017, was excluded from this analysis, as our automated counting algorithm could not fully account for the aggressive spread this sample shows in cell culture, leading to large syncytia covering the entire well (Fig. 4). Within each subgroup, none of the AA residues significantly distinguished between clinical isolates with low or high fusogenic activity, as determined by the Meta-CATS algorithm.

### Higher temperatures are associated with increased inactivation in all isolates

**Fig 5:**
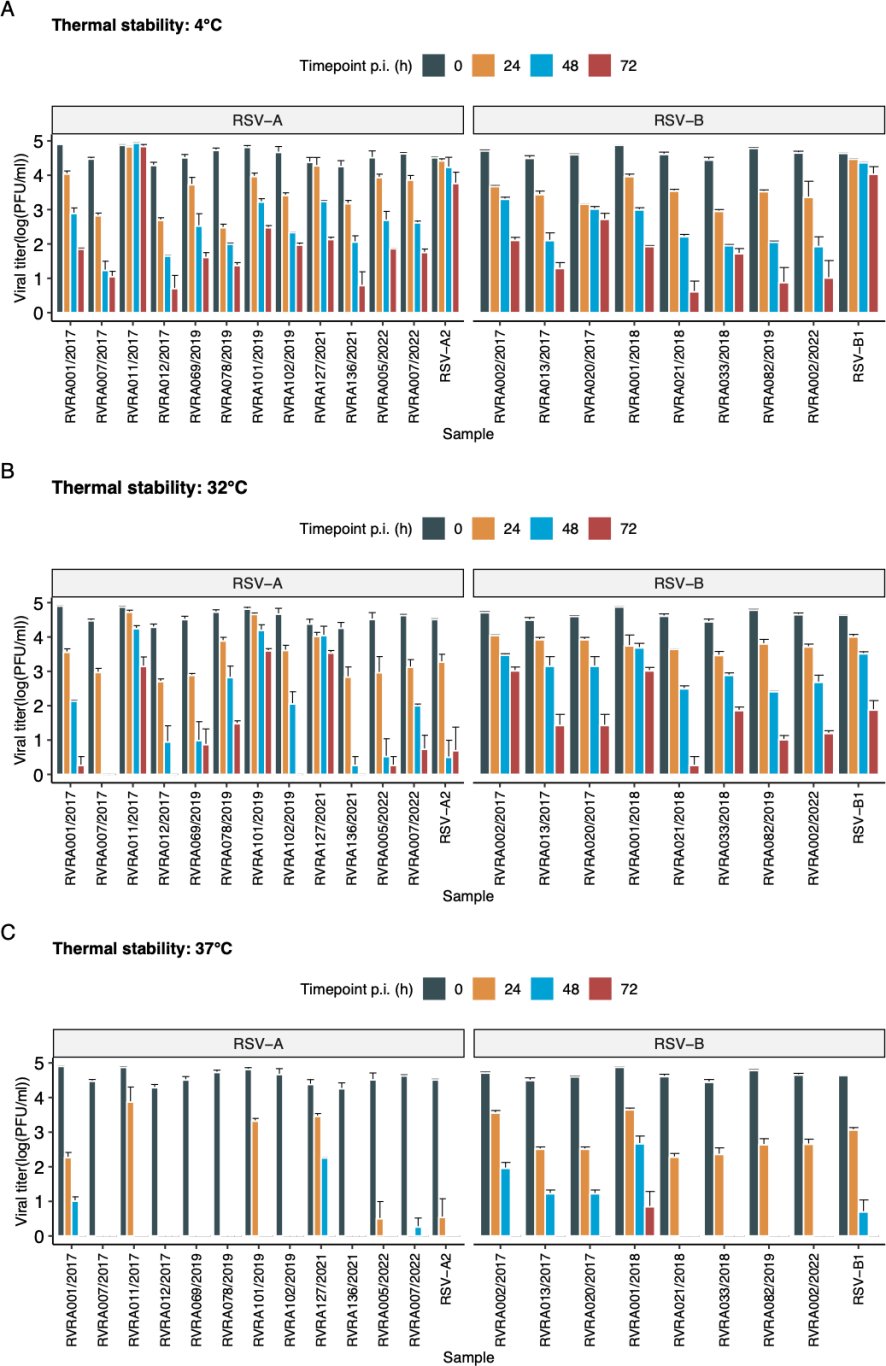
Evaluation of thermal stability. Thermal stability, expressed as the logarithm of the viral titer, was evaluated for each sample at three different timepoints and three different temperatures. Results are represented as mean ± 95% CI of three technical repeats. Thermal stability was compared using a linear mixed regression model with a random slope. For all viruses, inactivation rates varied significantly across the three tested temperatures (*P* < 0.001). At 4°C, the reference strains (*n* = 2) showed significantly higher stability compared to the clinical isolates (*n* = 20).

We assessed the thermal stability of our clinical and reference isolates after incubation in DMEM without FBS for 24, 48, and 72 h, at three different temperatures. The chosen temperatures were 4°C (representing refrigeration temperature), 32°C (representing upper airway temperature), and 37°C (representing general body temperature, including the lower respiratory tract). The rates of viral inactivation varied significantly across the three tested temperatures, with viral titers decreasing as the temperature increased for all viruses (*P* < 0.001; Fig. 5). At 37°C, titers rapidly dropped below detection limits for most viruses, while titers remained detectable at 4°C and 32°C. Intriguingly, RSV-B samples seemed more stable at 37°C compared to RSV-A samples, although this failed to reach statistical significance. Notably, HRSV/A/BEL/RVRA011/2017 demonstrated very high stability at 4°C, without any inactivation during the time of the experiment. Noticeable differences between reference strains (*n* = 2) and clinical isolates (*n* = 20) were observed at 4°C where both reference strains demonstrated higher stability compared to all clinical isolates, except the HRSV/A/BEL/RVRA011/2017 strain (*P* = 0.002). This difference was not observed at 32°C or 37°C.

**Fig 6:**
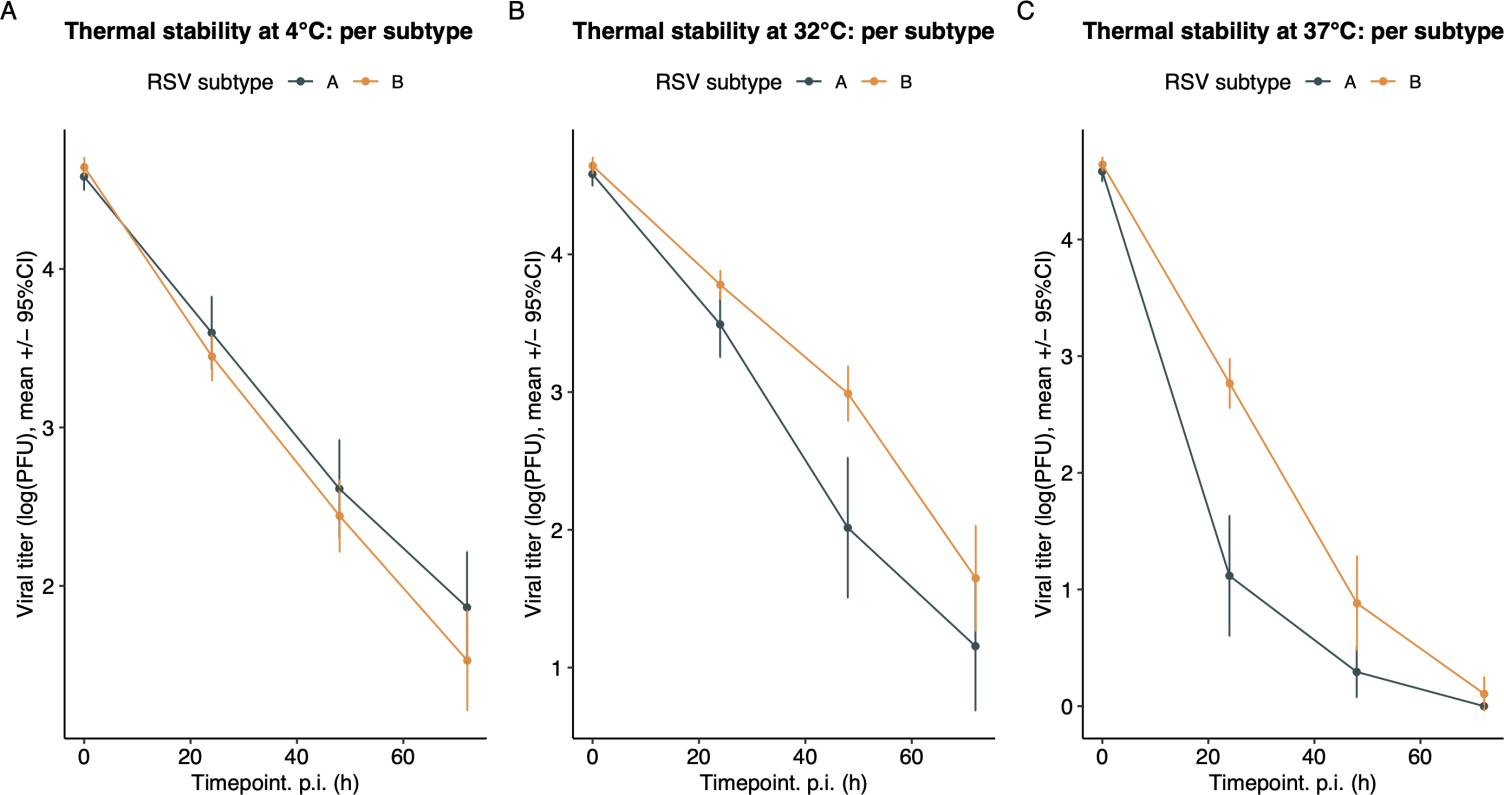
Comparison of the mean decay rate of both subgroups, at different temperatures, using a linear mixed effects regression (*n* = 20). Results are log transformed, because of heteroskedasticity, and expressed as means ± 95% CI. For each sample, three technical replicates were analyzed. Decay rates did not differ significantly between the RSV-A and RSV-B subgroups at any of the tested temperatures.

For each temperature individually, a linear mixed-effects model with random slope was fitted on the clinical isolates (*n* = 20), and the significance of an interaction term consisting of subgroup and timepoint was tested (Fig. 6). Although several RSV-B strains seemed more stable at 37°C, compared to RSV-A strains, this failed to reach significance (*P* = 0.07) for the overall subgroup comparison. Decay rates at 4°C and 32°C did also not differ between both subgroups (*P* = 0.59 and 0.20, respectively).

### F sequence variation seems insufficient to explain the divergent and often subgroup-dependent sensitivity to neutralization

**Fig 7:**
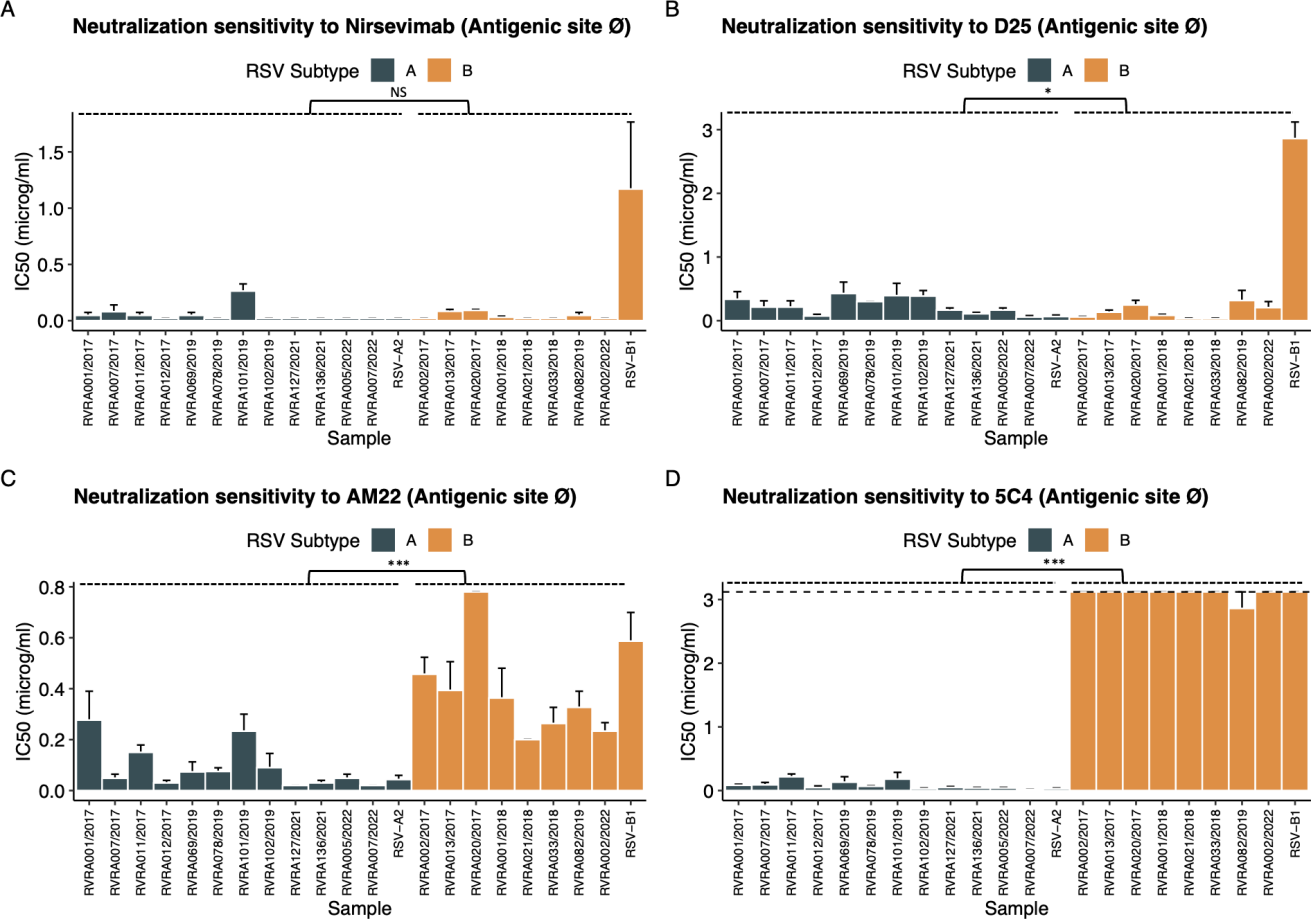
Neutralization sensitivity for mAbs targeting antigenic site Ø. Neutralization sensitivity is shown for each sample individually, expressed as the mean IC50 ± 95% CI. Error bars represent analyses conducted in three independent biological replicates. Comparisons between RSV-A (*n* = 12) and RSV-B (*n* = 8) subgroups were performed using the Wilcoxon test. Significant differences are indicated by * for *P* < 0.05, ** for *P* < 0.01, and *** for *P* < 0.001. NS denotes non-significant results.

With the anticipated widespread use of nirsevimab, we wanted to assess if our clinical isolates, which are representative of currently circulating RSV, differ in their sensitivity to nirsevimab and a panel of other mAbs with binding sites spanning the whole F protein. Looking at the site Ø mAbs, nirsevimab has a consistently low IC50 near or below the lower LOD (i.e., 0.05 µg/mL) across all isolates except the RSV-B1 reference strain (Fig. 7). Similar results were observed for D25, the mAb from which nirsevimab is derived. Interestingly, nirsevimab is the only mAb with comparable mean IC50 between RSV-A and RSV-B subgroups (0.05 vs 0.04, *P* = 0.44; Table 4). On the contrary, the AM22 and 5C4 mAb displayed an evident subtype-dependent sensitivity profile (Fig. 7; Table 4).

**TABLE 4:** Comparison of mAb sensitivity within and between RSV subgroups^*a*^

AS	mAb	IC50 (μg/mL), mean ± 95% CI				
		RSV-A (*n =* 12)	*P* value within RSV-A subgroup	RSV-B (*n* = 8)	*P* value within RSV-B subgroup	*P* value between subgroups
Ø	Nirsevimab	0.05 (0.049–0.055)	***<*** **0.001**	0.04 (0.040–0.043)	**0.001**	0.44
	D25	0.24 (0.229–0.245)	0.12	0.14 (0.131–0.144)	0.08	**0.01**
	AM22	0.09 (0.087–0.096)	**0.004**	0.38 (0.368–0.386)	***<*** **0.001**	***<*** **0.001**
	5C4	0.08 (0.079–0.087)	0.07	3.09 (3.081–3.094)	0.46	***<*** **0.001**
II	Palivizumab	1.94 (1.903–1.975)	**0.01**	1.13 (1.100–1.153)	**0.002**	***<*** **0.001**
III	MPE8	2.84 (2.813–2.864)	**0.01**	1.20 (1.155–1.247)	**0.001**	***<*** **0.001**
IV	101F	2.32 (2.281–2.355)	0.06	0.77 (0.731–0.800)	***<*** **0.001**	***<*** **0.001**
V	AM14	0.15 (0.142–0.162)	**0.02**	0.05 (0.050–0.055)	***<*** **0.001**	**0.02**
	Suptavumab	0.04 (0.034–0.037)	0.72	3.12 (3.120–3.120)	0.47	***<*** **0.001**
	CR9501	0.86 (0.822–0.893)	**0.002**	0.14 (0.134–0.143)	**0.02**	***<*** **0.001**

^*a*^ Mean IC50 (±95% CI) for each mAb was compared between clinical isolates (*n* = 20) from both RSV subgroups, analyzed using the Wilcoxon test, and within each subgroup, analyzed using analysis of variance (ANOVA). Experiments were conducted in three biological replicates.Significant values (*P* < 0.05) are indicated in bold.

The binding sites of these site Ø mAbs on the F protein largely overlap. Within the RSV-A subgroup, the binding sites of these mAbs were identical, and they all differed in only one AA residue with the RSV-A2 reference strain (K66E). Nevertheless, samples within this subgroup had significantly different IC50 values for nirsevimab and AM22. Of note, only one RSV-A sample demonstrated a 13-fold increase in IC50 (0.26 vs 0.02), compared to the RSV-A2 reference strain and a 5.2-fold shift compared to the mean (0.05) for all RSV-A clinical samples. For the other RSV-A samples (4/12), the fold increase compared to RSV-A2 was limited to four or less, which could be considered clinically irrelevant (35). Conversely, sequence variation was present within the RSV-B group, with 6/8 clinical isolates having the I206M:Q209R mutation, which seems associated with a twofold lower IC50 (0.04 vs 0.09, *P* < 0.001) compared to the two clinical isolates not carrying these mutations. The sequence similarity at site Ø between RSV-B1 and the clinical isolates, apart from these I206M:Q209R mutations, does not explain the substantially higher IC50 values observed for nirsevimab and D25 in the RSV-B1 reference strain.

**Fig 8:**
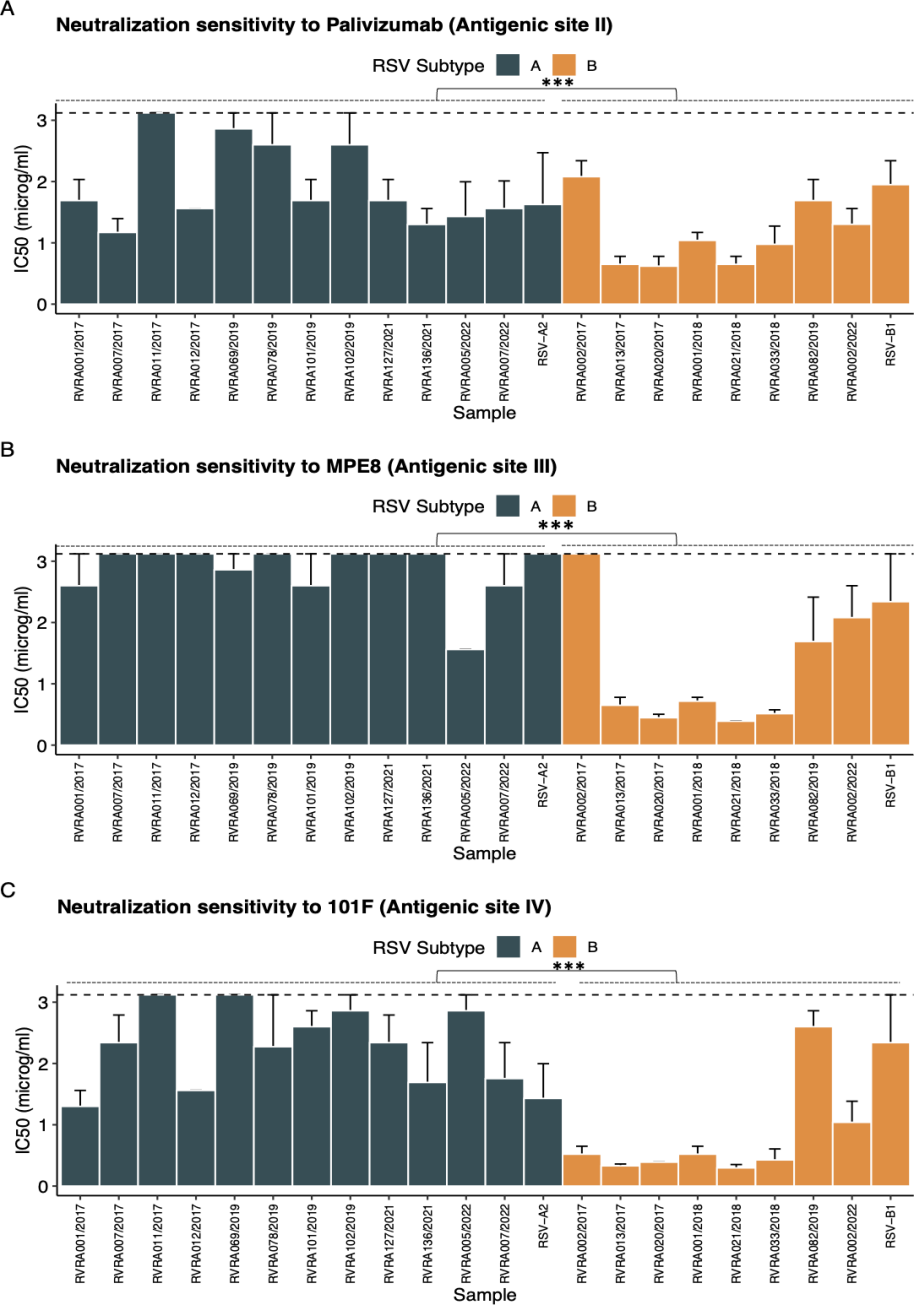
Neutralization sensitivity for mAbs targeting antigenic sites II, III, and IV. Neutralization sensitivity is shown for each sample individually, expressed as the mean IC50 ± 95% CI. Error bars represent analyses conducted in three independent biological replicates. Comparisons between RSV-A (*n* = 12) and RSV-B (*n* = 8) subgroups were performed using the Wilcoxon test. Significant differences are indicated by * for *P* < 0.05, ** for *P* < 0.01, and *** for *P* < 0.001. NS denotes non-significant results.

Mean IC50 for palivizumab, targeting antigenic site II, differs both between the subgroups and within (Fig. 8). However, the described binding site of palivizumab shows 100% sequence homology across all clinical and reference isolates, irrespective of the subgroup. One exception is a mutation at position 276 (S276T), adjacent to the palivizumab binding site, which is present in only one RSV-B isolate (HRSV/B/BEL/RVRA033/2018) that has a similar IC50 compared to the other B-isolates. The site III mAb MPE8 also shows considerable variation in mean IC50, but in this binding site, only one AA position differs between RSV-A and RSV-B samples, which is at position 305 and is considered a critical residue for MPE8 binding. Like palivizumab, the binding site of 101F in antigenic site IV has 100% sequence homology, which does not fully translate to the subgroup-dependent difference in sensitivity to neutralization by this mAb.

**Fig 9:**
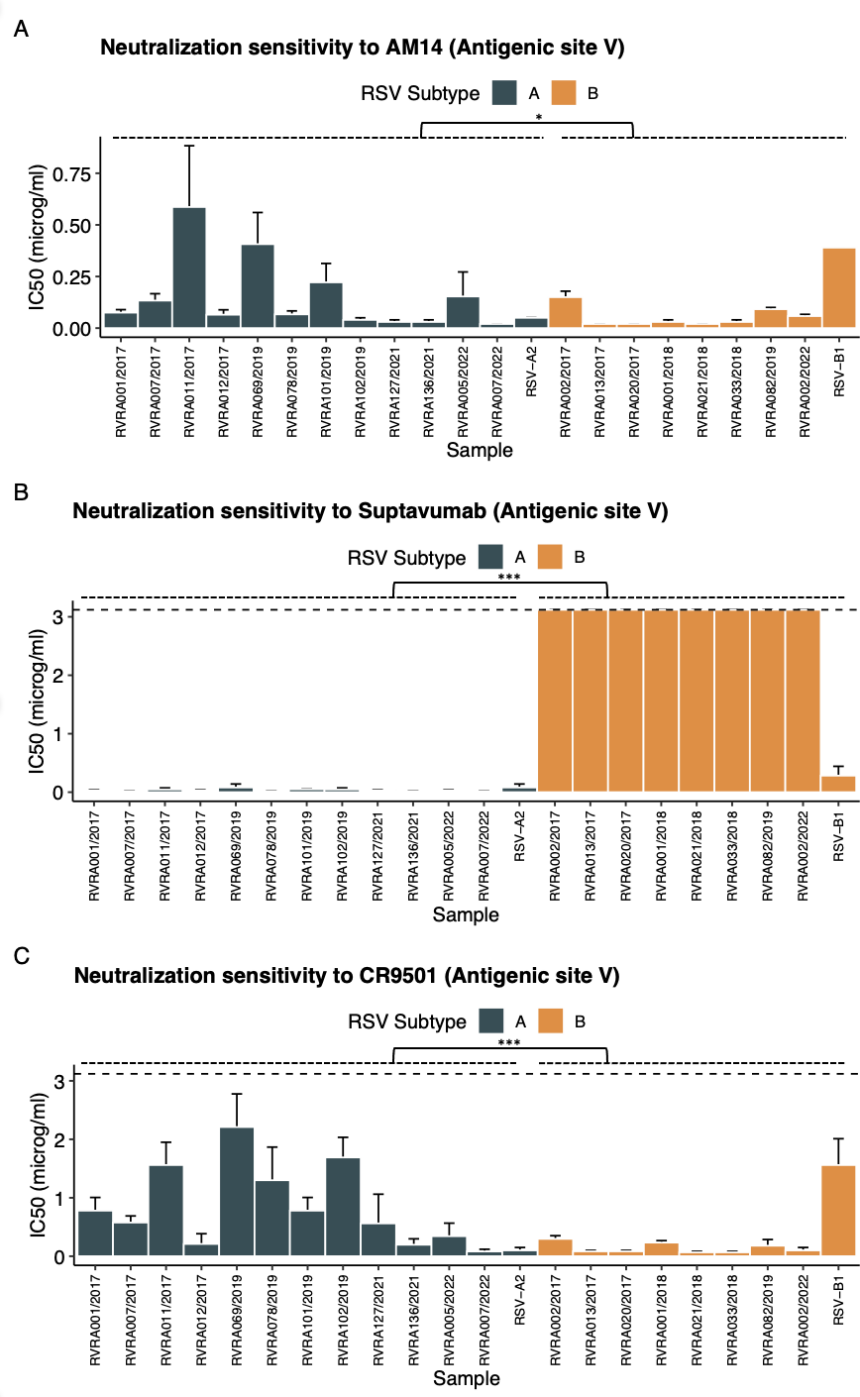
Neutralization sensitivity for mAbs targeting antigenic site V. Neutralization sensitivity is shown for each sample individually, expressed as the mean IC50 ± 95% CI. Error bars represent analyses conducted in three independent biological replicates. Comparisons between RSV-A (*n* = 12) and RSV-B (*n* = 8) subgroups were performed using the Wilcoxon test. Significant differences are indicated by * for *P* < 0.05, ** for *P* < 0.01, and *** for *P* < 0.001. NS denotes non-significant results.

Although both CR9501 and suptavumab bind with the same region within antigenic site V in the F1-subunit, CR9501 contacts substantially with the F2 subunit, which is not the case for suptavumab. This seems to be reflected in the observation that all clinical RSV-B isolates are resistant to neutralization by suptavumab, whereas they can be neutralized by CR9501, even at a lower mean IC50 compared to the RSV-A samples (Fig. 9; Table 4).

In conclusion, for the majority of mAbs, such as palivizumab and 101F, sequence variation within their binding sites is insufficient to account for the observed differences in sensitivity to neutralization. Notable exceptions include the 172/173 residues in the suptavumab-binding site, as well as the 201-residue variation in the 5C4-binding site, which seems a plausible explanation for the subtype-dependent sensitivity.

### Impact of *in vitro* phenotype on clinical disease severity

The observed phenotypical differences between clinical RSV isolates raised the question of whether these differences could, at least partially, account for the differential disease severity observed in patients infected with these isolates. When looking at all clinical isolates (*n* = 105), the ReSViNET score, admission rate, length of hospital stay, and need for supplementary O2 did not differ significantly between both RSV subgroups (*P* = 0.82, 0.91, 0.13, and 0.83, respectively), but the number of PICU admissions was higher in patients with an RSV-A infection (18 vs 7, *P* = 0.04). For those samples that were used for functional *in vitro* assays (*n* = 20), MSS and MSF did not correlate with disease severity, as expressed by the ReSViNET score (*P* = 0.62). However, growth rate, represented by the slope coefficient of a linear regression fitted to the viral titers at each timepoint, showed a weak positive correlation with the ReSViNET score (*R* = 0.26, *P* < 0.001). The slope coefficient of the growth curve was also significantly larger in patients who were hospitalized, needed intensive care treatment, or received supplementary oxygen (*P* = 0.003, < 0.0001, and < 0.0001, respectively). A point-biserial correlation showed, however, a statistically non-significant correlation between the slope of the growth curves and any of these disease severity-associated outcome measures (*P* = 0.31, 0.16, and 0.32, respectively). The decay rate of the samples at each tested temperature, represented by the slope coefficient of a linear regression fitted through the viral titers at each timepoint, did not correlate with any of the tested outcome measures for disease severity.

## DISCUSSION

For decades, RSV has been a major health issue, mainly in infants and young children, but it is also increasingly recognized as an important cause of ALRTI in the elderly. Effective treatments remain elusive; however, several new prophylactic agents were recently introduced, all targeting the RSV F protein. Widespread administration of these prophylactics is expected to have an important impact on RSV epidemiology. Nevertheless, significant knowledge gaps remain in our fundamental understanding of this virus. In this study, we set out to investigate F sequence variability and how this variability translates to an *in vitro* phenotype, using a set of recent clinical isolates. To the best of our knowledge, this is the first study to provide an in-depth analysis of a large set of clinical RSV isolates, combining both genomic and functional data, whereas other research often relies on laboratory strains or recombinant viruses.

RSV genotype diversity decreased over the last two decades, and since 2014, ON and BA have become the sole lineages detected for RSV-A and RSV-B, respectively (36). This historical classification system, based on the second hypervariable (HVR2) region of the G protein, has, however, proven to lack robustness, as neither the HVR2 fragment nor the entire G gene harbor sufficient phylogenetic signal to reliably construct phylogenetic trees (37). Additionally, the substantial heterogeneity in the antigenic surface of the prefusion trimer is unaccounted for in this classification (36). Therefore, we chose to employ the new classification system presented by Goya et al*.* earlier this year and used both G and F sequences to assign our isolates to the existing lineages (5, 38). This phylogenetic analysis revealed that, in our samples, RSV-A exhibits greater genetic diversity compared to RSV-B. RSV-A isolates demonstrated a broader range of lineages, with A.D.5 being the most prevalent, while RSV-B isolates were predominantly clustered in the B.D.4.1.1 lineage. In contrast, RSV-B strains showed more stability. It should, however, be noted that all but one of the RSV-B isolates date from the pre-COVID-19 period, which could explain the observed lower pairwise distance in RSV-B isolates in this study. The dominance of the RSV-A subgroup in the post-COVID-19 era in our regions agrees with other reports and correlates with a higher reproductive number for this subgroup (39, 40). The observed lower occurrence of RSV-B in recent years in our region, together with the genetic stability, raises the possibility that RSV-B strains might have lost the ability to escape the built-up herd immunity. Nonetheless, RSV-B outbreaks in regions such as the US immediately following the pandemic suggest significant regional variation, which seemingly contradicts this hypothesis (41). This highlights the importance of continued global surveillance of RSV to better understand its evolving patterns.

The high conservation of key antigenic sites on the F protein across both RSV-A and RSV-B isolates is crucial for the efficacy of current vaccines and monoclonal antibodies. The I206M:Q209R mutations we found in the nirsevimab-binding site of most of our B-isolates were associated with a 2.3-fold lower IC50, which corresponds with previous reports (42). It should, however, be noted that the clinical impact of such a small difference in IC50 is probably minimal. We also observed a mutation adjacent to the palivizumab-binding site (S276T) in one RSV-B strain. Although this mutation has not been described before, the N276S mutation in RSV-A and S276N in RSV-B has been reported. The N276S mutation in RSV-A strains was allegedly associated with decreased sensitivity to palivizumab; however, this was later contradicted (32, 34). The mutation found in our isolate also does not seem to impact neutralization sensitivity, but in a mouse experiment, mutations at this residue were shown to induce resistance to the mouse monoclonal antibody against antigenic site II, suggesting that the 276th AA could be susceptible to selection pressure (33).

Our analysis of growth kinetics and fusogenic capacity shows a clear subgroup-dependent *in vitro* phenotype, with RSV-A strains reaching higher viral titers after 72 h, with fewer, but larger syncytia compared to RSV-B strains. While other reports have drawn similar conclusions, these data are usually based on studies using prototypic strains or recombinant viruses, as opposed to the clinical isolates that were used here (43–45). Although the F protein is known to be responsible for fusion, the exact molecular determinants underlying these phenotypic differences remain largely unknown. Hotard et al*.* found that mutations at residues 79/191 and 357/371 in the RSV A2 line 19 F protein directly contributed to the fusion activity of this laboratory strain (46). However, in our study, we did not observe any variation at these residues. Similarly, Lawlor et al*.* demonstrated that an AA difference at position 66 (K66E) of the F protein was a major determinant responsible for growth and fusion differences seen between two recombinant RSV vaccine candidates (47). In our study, variation at this residue seemed, however, insufficient to explain the observed differences in fusogenicity between the clinical isolates, as all clinical RSV-A isolates carry a lysine at AA 66, which supposedly promotes fusion, as opposed to the reference strain RSV-A2, which carries a glutamic acid, which is thought to hinder fusion. No significant fusogenic differences were, however, observed between the clinical isolates and the A2 reference strain. In this study, we were thus unable to identify a clear molecular base for the distinction between highly fusogenic and poorly fusogenic strains. Intriguingly, our study identified a weak positive correlation between viral growth rate and disease severity, as measured by the ReSViNET score. Higher growth rates were also observed in hospitalized patients, as well as in those requiring oxygen supplementation or intensive care. One could hypothesize that a more rapid viral spread within the respiratory tract may be a stronger trigger for severe disease or overwhelm the immune system, hindering an effective immune response and subsequently exacerbating symptom severity. Furthermore, faster replication rates might be associated with increased tissue damage and therefore a poorer prognosis. Alternatively, underlying host factors, such as immune system dysfunction, could facilitate more rapid viral replication while simultaneously predisposing individuals to more severe disease outcomes. However, the limited sample size used for functional testing restricts the ability to draw definitive conclusions.

RSV F consists of two conformations: the metastable pre-F conformation, which readily transitions into a thermodynamically stable post-fusion conformation. It has been shown that this transition can be induced by sustained elevated temperatures (48). Additionally, it has been hypothesized that the stability of an RSV isolate is correlated with the amount of virus that is present in pre-F conformation, and RSV samples with high amounts of pre-F are more resistant to heat inactivation (49). Although previous data suggested that AA residues 357 and 371 modulate viral resistance to thermal inactivation, none of our isolates showed a substitution at these residues (49). We observed significantly different decay rates between each of the tested temperatures, with increased temperatures being associated with increased decay. This is also reflected in several short-term storage protocols, which advise pre-processing storage of isolates at 4°C. However, a recent Japanese study found, contrary to common understanding, an unexpectedly greater loss of viability of clinical RSV isolates upon short-term storage at 4°C than at room temperature (50). Furthermore, the stability of the HRSV/A/BEL/RVRA011/2017 isolate at refrigeration temperature is remarkable and could be advantageous, for instance, for a candidate live attenuated vaccine. Although the modeling was not statistically significant, RSV-B clinical isolates demonstrated remarkably higher stability at 37°C. In the context of a febrile child, where body temperatures rise above 38°C, this could hold clinical relevance. Several research groups have already investigated the influence of subgroups on clinical presentation or disease severity but failed to generate unequivocal conclusions (36). Nevertheless, this finding warrants further investigation, as this might imply prolonged illness duration and prolonged viral shedding in infections with the RSV-B subgroup.

The statistically significant variation in sensitivity to neutralization among isolates of the same and different subgroups might not always translate to a clinically detectable shift in susceptibility if the fold increase was limited (i.e., lower than 5) (35). However, it emphasizes the necessity for ongoing surveillance to ensure the effectiveness of current and future prophylactics. Surprisingly, altered sensitivity to neutralization can sometimes, but not always, be explained by differences in AA sequence of the concerned binding site. For instance, the subtype-dependent neutralization profile of 5C4 is likely due to its requirement for a positively charged residue at RSV F position 201, which is absent in current RSV-B strains (51). A similar subtype-dependent sensitivity is observed for MPE8, with the 305 residues being the only divergence between both subgroups (LEU vs ILE). Although this residue has been deemed critical for mAb binding, it seems unlikely that this AA change, with both belonging to the same group, is solely responsible for the observed differences. In contrast, for some mAbs, the known AA sequence of binding sites is insufficient to explain the differences in mAb sensitivity, as is exemplified by the 100% sequence homology in the palivizumab and 101F epitopes despite clear differences in neutralization sensitivity. For 101F, this might be due to substantial contacts with additional surfaces outside the linear epitope (52). Furthermore, not all epitopes are linear, as is the case for AM14, which binds with a quaternary trimer-specific epitope that is sandwiched between antigenic site IV and V. Additionally, since the HRSV/A/BEL/RVRA011/2017 isolate demonstrates a relatively higher mean IC50 for various mAbs—except the site Ø mAbs—and greater thermostability at 37°C, it can be hypothesized that the stability of the pre-F conformation and thus the relative quantity of pre-F conformation in a virus could impact the neutralization of that virus by a given mAb. Lastly, it is also possible that F interacts with other RSV proteins, such as the G protein, and these interactions could impact sensitivity to neutralization. The latter implies that using recombinant viruses based on F sequence variation alone might be insufficient to detect and explain all variations in mAb susceptibility. Taken together, this detailed analysis using a panel of mAbs with different epitopes across the F protein in clinically relevant RSV isolates suggests that AA sequence is not always sufficient to explain the observed sensitivity to neutralization, and other factors outside the specific epitope, or even outside the F protein, might be implicated.

A few limitations must be acknowledged. First, the employed virus propagation method might have resulted in the selection of a relatively homogeneous virus population that may not fully represent the diversity found in a clinical sample. Despite efforts to mitigate this by combining multiple wells at each propagation step, the selection of a dominant clone cannot be excluded. Furthermore, some SNPs were observed at a low frequency, sometimes even in only one isolate. This might be attributed to sequencing errors, although this risk was minimized by performing sequencing in duplicate. Alternatively, these could represent mutations that were introduced due to adaptation to cell culture conditions, although it seems unlikely that such changes would occur within only three passages. Another possibility is the presence of multiple subpopulations in one sample, of which the minor population became dominant due to the selection pressure imposed by cell culture passaging (34). In six clinical samples, we indeed found one or two AA differences in the F protein between P0 and P3, none of which occurred in the binding epitopes of the tested mAbs. Although the significance of these discrepancies is unclear, this does not compromise our finding that a different *in vitro* phenotype was observed between various viral isolates with dissimilar F sequences.

Furthermore, for phylogenetic analysis, it is becoming evident that the second hypervariable region in the G gene is insufficient. Although whole-genome sequences are ideally used for molecular epidemiology studies, this approach is labor intensive and expensive. Therefore, we chose to use only the full G and F sequences here, as the recent consensus considers this sufficient to reproduce the topology of the RSV phylogenetic tree (38). Lastly, it is important to note that the sample population studied here is rather not representative of the overall population susceptible to RSV, as mildly ill patients are underrepresented. Consequently, making assumptions about this population might introduce selection bias. Additionally, the sample size used for functional analysis was relatively small. However, these samples were specifically selected to encompass all variation present in our samples, without overrepresentation of similar or even identical F sequences. Although we did not find any relevant correlation between the F AA sequence and clinical disease severity in our study population, we observed a statistically significant but weak positive correlation between the growth rate of our samples and the observed disease severity in patients from whom the samples were collected. These results should, therefore, be interpreted with caution and confirmed in larger studies.

In conclusion, our analysis shows that genomic variability in the F protein among contemporary clinical RSV isolates is limited. Despite these limited genomic differences, large phenotypic differences can sometimes be observed in traits that can be linked to the F protein, such as syncytium formation, virus stability, and neutralization by RSV F-specific mAbs. Moreover, it is increasingly evident that commonly used reference strains are no longer representative of currently circulating RSV isolates, a finding reinforced by this study. These findings underscore the importance of continued surveillance of RSV genetic and antigenic variation to inform vaccine development and antiviral strategies. While current prophylactic measures appear effective and are anticipated to drastically impact RSV epidemiology, the observed diversity in the spread and stability of RSV strains in our study necessitates ongoing monitoring to adapt to evolving viral variants. Furthermore, future research should focus not only on molecular surveillance but also on the phenotypical characterization of recent and clinically relevant RSV isolates.

## Data Availability

The sequencing data are publicly available at National Center for Biotechnology Information (NCBI) with the identifiers PQ410466–PQ410666.

## SUPPLEMENTAL MATERIAL

Fig. S1 msphere.00860-24-s0001.docx Phylogenetic tree, RSV-B.

Fig. S2 msphere.00860-24-s0002.docx Phylogenetic tree, RSV-A.

Fig. S3 msphere.00860-24-s0003.docx Comparison of MSS and MSF between individual samples.

ASM does not own the copyrights to Supplemental Material that may be linked to, or accessed through, an article. The authors have granted ASM a non-exclusive, world-wide license to publish the Supplemental Material files. Please contact the corresponding author directly for reuse.
