# Supplementary material for: Functional implications of respiratory syncytial virus F sequence variability: a comparative analysis using contemporary RSV isolates
Source: mSphere. 2025 Apr 14;10(5):e00860-24. doi: 10.1128/msphere.00860-24 (PMC12108066; doi:10.1128/msphere.00860-24)
Supplement: Fig. S1 — Phylogenetic tree, RSV-B. [file msphere.00860-24-s0001.docx]

S1 Fig. Phylogenetic tree of RSV-B isolates, constructed by maximum likelihood method in IQTREE and visualized with figtree. Clinical isolates are represented in red, together with reference strains (Goya et al) in black. Different colors represent different lineages: B.D.4.1 in light blue, B.D.4.1.1 in dark blue, B.D.E.2 in purple, B.D.E.3 in pink.
