# Supplementary material for: Functional implications of respiratory syncytial virus F sequence variability: a comparative analysis using contemporary RSV isolates
Source: mSphere. 2025 Apr 14;10(5):e00860-24. doi: 10.1128/msphere.00860-24 (PMC12108066; doi:10.1128/msphere.00860-24)
Supplement: Fig. S3 — Comparison of MSS and MSF between individual samples. [file msphere.00860-24-s0003.docx]

S3 Fig. Comparison between mean syncytium size (A) and mean syncytium frequency (B) between individual clinical isolates. Data are represented as means +/- 95 CI of three biological repeats.
